# Weaving a new blanket together: lessons on compassionate leadership and engagement from a virtual regional summit on early childhood wellness in northern communities of British Columbia, Canada

**DOI:** 10.1186/s40900-022-00391-5

**Published:** 2022-10-20

**Authors:** Erica Koopmans, Lisa Provencher, Lauren Irving, Caroline Sanders

**Affiliations:** 1 School of Health Sciences, University of Northern British Columbia, 3333 University Way, Prince George, BC V2N 4Z9 Canada; 2 Research and Knowledge Exchange, First Nations Health Authority, West Vancouver, Canada; 3 School of Nursing, University of Northern British Columbia, Prince George, BC Canada; 4 Northern Health, Prince George, BC Canada

**Keywords:** Compassion, Systems thinking, Early childhood, Engagement, Leadership

## Abstract

**Background:**

Early childhood is a critical period of development for infants, young children, and their families. An array of services, programs, and interventions exist to support families during this life stage, often delivered by a diverse range of professionals. Overlap in early years services exists between healthcare, social care, childcare, education, and not-for-profit organizations. Such diversity in services has the potential to add a rich experience to early childhood development, or without collaboration, widen service gaps, risking providers’ ability to meet the needs of families.

**Methods:**

In northern British Columbia (BC), Canada, a group of individuals came together to approach building relationships and engagement across sectors in early years services using compassionate systems leadership (CSL). A virtual summit was hosted with early childhood service providers including peer support workers and parents/caregivers using a hybrid model of pre-recorded asynchronous sessions combined with a live workshop. The purpose of the event was to find common ground, celebrate local success, and build understanding of how to work collaboratively across the region to identify and address early years priorities.

**Results:**

The event was successful in engaging 121 providers across early years services from a broad geographic region. Applying CSL principles for engagement allowed the team to examine how all partners could address silos in early years services across northern BC. Using a reflexive thematic approach, four key themes were identified at the Summit: (1) early years services are a patchwork but there are dreams of weaving a new blanket together, (2) an ideal model of service is family-centred and inclusive, (3) all sectors are needed at the table, and (4) compassion is the thread that weaves this work together.

**Conclusions:**

The application of CSL principles can be used to guide engagement and develop supportive spaces for open conversation about creating systems change. In facilitating a space that allowed for vulnerability and relational ways of engaging across sectors we discovered commitment and a willingness for those present to consider new ideas and partnerships that would allow for greater integration of early years services in northern BC.

## Plain English summary

For children aged zero to eight years old there are a wide range of community services geared to supporting health, pre-school education, and parenting skills and knowledge. Getting the right services at the right time in early childhood can make a major difference to a child’s health and development and influence their physical and mental health outcomes into adulthood. We know there are individuals from a wide range of sectors working with families during early childhood. Sectors include the health system, education, government ministries, childcare centres, and community programs. Despite working with many of the same families, each sector’s services are often seen as separate which means that the opportunity to develop shared, family centred goals can be missed. In northern British Columbia, Canada we hosted a virtual event with 121 people who work in roles that support children between the ages of zero to eight and their families living in smaller, more rural communities. Using a blend of pre-recorded videos and a live online workshop we drew together a community of providers to learn about the work they are doing. Specifically, we were interested in exploring ways in which we can improve working together to support positive early childhood experiences for children and their families.

## Background

The early years are traditionally considered the stage of development in childhood from birth to age eight [1]. This period in early childhood is a critical time for brain development, self-concept, and identity formation [2]. The effects of the early environment, both positive and negative, are long-lasting and critical in future life-outcomes, with links between early-life circumstances, school performance, adult literacy, health status, and life expectancy [3–5]. Research suggests that one of the best ways for us to improve the health of the whole population is to focus on evidence-based policies and interventions that optimize both early childhood health and development, as well as early years education [5]. Evaluation demonstrates that early childhood services, when delivered effectively, can result in a wide range of positive long-term health and wellbeing outcomes for children and families [6–11]. A diverse range of professionals from many sectors such as health, social care, childcare, education, and not-for-profit organizations are engaged in working with children and families in the early years. Such diversity has the potential to add a rich experience to early childhood development and address interrelated medical, social, behavioural, educational, and financial needs of families. However, it can also lead to service gaps and wider systems failure when coordination of care does not occur [12, 13].

To break down barriers that exist in early years services, we must purposefully look at how we work together. Building relational ways of engaging within a model of compassion can allow for individual service models to explore congruence, respectful of the unique aspect each service brings to early childhood. While individual programs can offer positive and culturally appropriate engagement, working in partnership within the context of communities can help to identify interrelated and integrated approaches that balance time, resources, and energy [14]. When taking a multi-sectoral approach, the collective impact can manifest in different ways. The process and outcomes of collective impact are emergent rather than predetermined; the necessary resources and innovations are often available but are not always recognized; learning is continuous; and adoption occurs simultaneously across a wide range of organizations [15]. Community connection and awareness of early years programming across all sectors can support financial strategies that have been noted to limit programming [14] and help address overuse, misuse, and underuse of services in ways that maximize the potential in early years work. Overall, the potential benefit in focused attention and prevention efforts in the early years is greater if shared priorities and outcomes can be determined between sectors.

### Geographical context

Northern British Columbia (BC), Canada is a large geographical area with over 30 municipalities and approximately 54 First Nations’ groups [16]. Each community has its own set of challenges in terms of early childhood health and development, however across the region we have shared concerns regarding poor outcomes on indicators of early childhood health and wellbeing. In BC, the early development instrument (EDI) is completed by kindergarten teachers for each student in their class and data is reported regularly by the Human Early Learning Partnership to provide insight on the health and development of children. The EDI collects data on five domains of development: physical health and wellbeing, social competence, emotional maturity, language and cognitive development, and communication skills [17]. Vulnerability on one or more of the five scales has consistently increased in northern BC. In the most recent set of data collected, 37% of children entering kindergarten were reported as vulnerable on one or more of the scales [18]. In some communities in northern BC this rate reaches nearly 50% [18]. Despite educational materials, tools, programs, and other resources that exist to support families and service providers, we are not making measurable and sustained changes in these outcomes.

### Compassionate systems leadership and innovation

Aware of the current early childhood context in northern BC, our team came together to explore building community among early years service providers in the region. The team was interested in using Compassionate Systems Leadership (CSL) to bring individuals across sectors together to learn as a community, nurture local partnership systems, and to build capacity and infrastructure for early years collaboration. Our small group had learned about CSL through a year long program delivered in 2018 by the Human Early Learning Partnership. We had come to value connections, learning, and resources as interconnected CSL antecedents that we could creatively apply in addressing early years priorities in our communities.

The CSL framework looks to develop capabilities and knowledge that strengthen the capacity of both individuals and collectives to effectively progress systems change initiatives [19]. The three key components are personal mastery (self-leadership), interpersonal skills (leading relationally), and systems thinking (connections between individuals, groups, and the wider community) [19]. Practicing CSL involves personal journey, starting with inner change and then working towards creating outer change that can support new ways of thinking. Often this combines mindfulness at a personal level, and respect in recognising leadership potential in others at every level within a system and across systems. CSL embraces continuous learning to develop knowledge and skills in ways that can be applied to practice. West et al. report four key elements that need to be in place across systems for innovation to occur under compassionate leadership. These are (1) inspiring vision and strategy, (2) positive inclusion and participation, (3) enthusiastic team and cross-boundary working, and (4) support and autonomy [20]. These CSL principles foster an environment that can provide space to think differently, unincumbered by system barriers and prior beliefs of how organizations should act, which in turn allows individuals and groups to advocate for systems change and innovation. The interest in applying CSL to guide this work evolved into an initiative titled Sharing to change Early childhood Experiences and promote healthy Development in northern BC (SEED BC) [21]. The launching point for this work was focused on connection and engagement, which resulted in the SEED BC Summit.

### Objectives

The purpose of the Summit was to bring together child and family sectors (health, education, social care, and not-for-profit) to build connections, learn about work happening in communities and to celebrate successes. We aimed to find common ground and develop understanding of shared needs and priorities in the early years across northern BC using a CSL approach. The aim of this article is to share a descriptive summary of the Summit and key learnings from this multi-sectoral engagement approach.

## Methods

### Planning the summit

A regional advisory committee was convened with diverse representatives to guide the Summit planning. Representatives included: researcher and research trainees, population health and child health leaders from the regional health authority, nurse practitioner, family physician, child and adolescent psychiatrist, early childhood educators and childcare providers, municipal government planner, administrators from child development centres, parent peer support workers, and program leads from an Indigenous agency and not-for-profit organization. The advisory committee was engaged in all aspects of the Summit planning. Such a diverse committee was possible because of existing relationships between some members and the prior shared learning in compassionate leadership. These relationships and training helped us reach out broadly to others in our network who we knew shared our belief in the value of early childhood and allowed us to develop friendships that have lasted in building this advisory committee.

The Summit was initially planned as a face-to-face event in April 2020, however due to the COVID-19 pandemic, it was redesigned and remained a free event that took place virtually in February 2021 and ran over five days. It was delivered using a hybrid model of pre-recorded asynchronous sessions combined with a live full day workshop on the final day. Participants were offered the option to register to access asynchronous sessions only, or asynchronous sessions combined with the live workshop. The workshop was split into a morning session and afternoon session. The morning was comprised of a live one-hour presentation on CSL to introduce participants to the framework followed by a panel discussion. The panel membership consisted of eight individuals, three of whom were Indigenous, and was comprised of individuals who had received training in CSL and integrated this into their way of working in the early years. This was followed by facilitated breakout groups and reflection sessions in the afternoon. Woven throughout the day were mindfulness practices including breathing breaks, reflection questions, and moments for individual celebration of the work participants are a part of in their community. While the Summit was focused on northern BC and targeted individuals living and working in this region, registration was inclusive and allowed others who were interested to participate.

Northern BC has a small population, yet it is home to approximately 35.6% of the province’s Indigenous population [22]. Across all services in the region care providers work with Indigenous children and families in the early years. Paying careful attention to ensure inclusion and representation of people and communities, in particular Indigenous partners, was a critical aspect of our planning. In Canada, supporting Indigenous children and families in the early years requires developing approaches that are rooted in Indigenous cultures and knowledge, and focusing holistically on protecting language, identity, and rights [23]. As care providers and researchers, we are personally and professionally committed to the Calls to Action of the Truth and Reconciliation Commission of Canada [24] and acknowledge that the current gaps in early childhood outcomes between Indigenous and non-Indigenous populations are a direct result of colonization, government policies, systemic inequities and racism [25, 26]. It is essential that equitable and culturally appropriate early childhood programs and services are developed in partnership with Indigenous families and communities [24, 27]. In planning the Summit, we committed to using the platform to amplify the voices and cultural practices of Indigenous partners and presenters, creating a space to share their expertise, knowledge, experience, and guidance as it relates to early childhood and to incorporate and normalise the use of Indigenous languages into the event. The Summit embraced both Indigenous and Western pedagogies, acknowledging that both are necessary to improve health and wellbeing of children and families in our region.

### Engagement approach

Preceding and throughout the Summit week we created multiple avenues for engagement through sharing of stories, experiences, and ideas, and space for reflective feedback. In advance of the Summit, we leveraged the Thought Exchange platform to ask individuals, “*What is needed to improve early childhood health, education, and wellbeing in your community?”.* Thought Exchange is a crowd sourcing platform used to engage groups by gathering ideas and focusing on alignment and prioritization of these thoughts. An Exchange is created asking an open-ended question to which participants confidentially provide their responses. Responses are then randomized so participants can objectively rate other responses. During the Summit week an online engagement space was created using Padlet which served as a "community whiteboard". It was primarily used as a space for participants to connect, share comments, learning from the day, and resources. To facilitate discussion, each day a question or engagement suggestion was posed to encourage participants to share their thoughts for example, “*how do we keep the early years near the top of the pile when we are faced with many demanding priorities?*”. Finally, facilitated breakout groups took place during the virtual workshop. Participants were divided into six small groups led by facilitators equipped with a set of discussion questions the to guide the conversation. The facilitator guide was developed with support from the Centre for Teaching and Learning at the University of Northern British Columbia and reviewed by the Summit advisory committee. Facilitator training was provided pre-Summit with the planning team and their assigned note taker. This training covered a range of topics in preparation for the breakout groups including ground rules (such as muting microphones when others are talking, using the raise hand function to indicate they have something to add, waiting ones turn), maintaining anonymity in dialogue in the small groups, and fostering inclusion in the conversation. The first breakout focused on ‘Dreaming with partners about what could be’ and discussion questions included, “*What would an ideal model of health and wellness in the early years (0–8 years) in northern BC look like?*” and “*Describe an example of what positive partnership looks like in your community. How did you get there?*.” The second breakout was a focused discussion on understanding and applying compassionate leadership. Discussion questions included “*how do you understand compassion as a platform to build community?*” and “*what are you motivated to change or continue in your community?*”. Each group had a note taker, who also had pre-Summit training and a guide that outlined ethical principles in documenting the discussion. Following each breakout session, a member from each group reported back to the larger workshop audience the key takeaways from their discussion. This was an opportunity for reflection, exploring shared findings across groups, and learning about differing or challenging ideas. At the end of the workshop, space was reserved for real time evaluation and feedback as the group reflected on the day, specifically what had been successful and valued in the Summit approach and what did not work. A short evaluation survey was distributed to all asynchronous and live participants via email the week following the event, followed by a postcard mailed to participants in the month after the event.

### Analysis

Descriptive statistics were used to summarize participation and Thought Exchange data. A reflexive thematic approach was selected for analysis of all qualitative data. This iterative approach to analysis allowed for data immersion and conceptualization of shared meaning to support interpretation of the data [28]. Thematic analysis was conducted by three team members with backgrounds in health service planning and public health (EK), paediatric nursing and qualitative inquiry (CS), and early childhood education (LP). Team members first familiarized themselves with the data by reading and re-reading field notes from each small discussion group. Initial themes were generated by each team member and then reviewed and discussed as a team to reflect on the meaning, interpretation, and naming. Each team member brought a unique perspective from their experience that supported reflexivity through rich discussion during theme development, and interpretation of meaning from varying perspectives of health, education, and social care. These themes were then discussed and mapped in relation to the four key cultural elements that support innovation to occur under compassionate leadership: (1) Inspiring vision and strategy, (2) Positive inclusion and participation, (3) Enthusiastic team and cross-boundary working, and (4) Support and autonomy [20].

## Results

### Participation

One hundred and twenty-one individuals registered to participate in the Summit with representation across child and family serving sectors. 96% of participants were from BC, with 90% living and working in the northern region of the province. Table 1 presents the characteristics of Summit participants.

**Table 1:** Characteristics of Summit participants

Self-identified participant demographics	*n* = 121 *n* (%)
*Region of community where participant primarily lives and works*	
Northern interior BC	71 (59)
Northwest BC	37 (22)
Northeast BC	11 (9)
Other region of BC	7 (6)
Outside of BC	5 (4)
*Primary role of Summit participants*	
Educator (e.g., early childhood educator, teacher)	36 (30)
Early childhood program coordinator or facilitator	20 (17)
Healthcare provider (e.g., physician, nurse practitioner, nurse, physiotherapist)	15 (12)
Peer support worker, outreach worker, or parent/caregiver	12 (10)
Mental health clinician	11 (9)
Manager, administrator, or policy maker	9 (7)
Student	5 (4)
Childcare provider	5 (4)
Researcher	4 (3)
Behaviour analyst	2 (2)
Social worker	2 (2)

During the week there were 863 video views of asynchronous sessions from 142 unique viewers. The most viewed asynchronous sessions were focused on trauma and childhood development; the regional health authority’s approach to early childhood services; and an overview of supporting families in communities of compassionate practice. The live full day workshop had 51 participants and 10 facilitators. Completion of the follow-up evaluation survey was limited (*n* = 9).

### Thought exchange

**Fig. 1:**
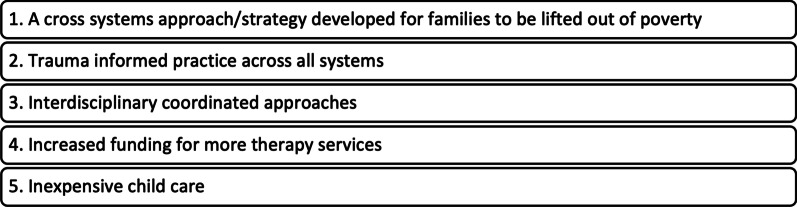
Top ranked needs for early childhood health, education, and wellbeing in northern BC communities

Forty-eight participants submitted a total of 71 key thoughts via the Thought Exchange platform to the question “*What is needed to improve early childhood health, education, and wellbeing in your community?”.* 643 ratings were provided, and the top five ranked needs for early childhood health, education, and wellbeing are presented in Fig. 1. These five needs in conjunction with the learnings from the Summit build an understanding of needs and priorities in the early years across northern BC.

### Summit experience

The Summit welcoming was led by a Lheidli T’enneh Elder in the Dakelh language. Our commitment to using the Summit platform to give space to listen to the voices of Indigenous partners resulted in 48% (10/21) of presentations being delivered by Indigenous leaders or focused on presenting partnered research or initiatives with Indigenous populations. This included presentations on northern Indigenous pedagogy; Indigenous perspectives on fatherhood and parenting; experiences of Indigenous caregivers who have children with attention-deficit/hyperactivity disorder; and the delivery of trauma and violence-informed early intervention with Indigenous families and children. Further, many leaders from northern Indigenous organisations presented at the Summit covering a range of topics e.g., mental health access and support (Native Friendship Centre) and primary health care (Indigenous health service organization). Overall participants expressed excitement at having the opportunity to convene with others from across northern BC to focus on early childhood wellbeing. There was appreciation for the space to reflect on the knowledge being shared and to learn about what was happening in other communities around all aspects of early childhood, specifically, what had been successful elsewhere, or attempted with lessons learned. There was also appreciation for a virtual space that supported equitable access to participate regardless of geography. Participants reported that the asynchronous delivery and engagement opportunities were an effective approach given the COVID-19 pandemic. Recommendations for improvement included a time limit for asynchronous presentations to keep them brief and to split the full day virtual workshop into two half day sessions.

### Summit learnings

#### Inspiring vision and strategy

*Early years services are patchwork but there are dreams of weaving a new blanket together.* Participants described the current state of early years services as fragmented, delivered in patches or pockets of work, resulting in limited or inconsistent family support. Parents and primary caregivers were noted to be the relational ties between pockets of care, often left trying to weave a blanket of care to support themselves as a family. Reflecting on the experiences of trying to support families in rural and small urban communities in northern BC, participants expressed that there needed to be a “new weaving of the blanket.” There was interest in greater cross-sector communication, coordination, and collaboration, and in the development of a more cohesive or integrated approach to supporting families and children in the early years. The new blanket was described as a holistic, multidisciplinary team that could result in a “seamless service.” Many described a hub model or a one-stop shop being an ideal approach for provision of cross-sector supports from pregnancy through to eight years old. The proposed hub approach was described as more than a physical space and drew on the value of building community relationships and networks of family voices, as well as physical spaces. Participants provided examples of some communities already undertaking this approach in northern BC. This hub model was highlighted by several Indigenous participants whose communities were moving forward with this approach. They described the hub as a place for to community to gather and the importance of Elders being included in this holistic work. One participant described in their community they are developing a wellness centre attached to the current health centre where Elders will be present to provide support and guidance to families. They described how Elders will be helping to make decisions within the new centre that will foster family preservation and support.

#### Positive inclusion and participation

*An ideal model of service is family centred and inclusive.* Children and families were central to all discussions. An ideal model of service was described as family centred and inclusive, unpacking what this meant for participants was a critical dimension of the workshop process. With support from the facilitators, participants explained how they had a deep understanding of families, developed through community connection, trust, and compassion. Participants offered examples that evidenced how families demonstrate experiential ‘knowing’ as experts framed around their family's needs. Such experiences allowed them to set their goals and not become hidden, forgotten, or lost within the present system of early years work. As described by one participant *“trusting that families in communities, no matter [the] type, know their needs – helps them to interpret, share their knowledge and grow and build upon that.”* Participants also emphasized that services need to be culturally responsive and include options for the diversity of primary caregivers in our communities including fathers, grandparents, aunts, uncles, and foster or adoptive parents. Participants identified there are often gaps in supports for these specific individuals as caregivers in early childhood, particularly fathers. Any system that is co-created, adopted, or adapted must be driven by the community and have the voices of the families and the children involved - at its heart, paying specific attention to cultural sensitivity and support for diversity, accessibility, and inclusivity. This can be accomplished in many ways. For example, one participant described how a field survey took place in their small community asking about the current strengths of the community, resources available, and the existing gaps. These results were then put into a booklet that was available to decision makers across sectors to hear from the community what was missing and where change could be made from the community perspective. They described that sometimes excuses are made such as *“we’re in the north and are missing so many things – the thing is we’re always going to be in north – so having individual and community areas for action is useful”.* This was one shared example of how family and community voices can be used but families need to be engaged at all stages when decisions are made around approaches and actions to drive change.

#### Enthusiastic team and cross-boundary (multi-sectoral) working

*All sectors are needed at the table. *Participants acknowledged the current demands on health providers due to the changing and ongoing nature of the COVID-19 pandemic, however, participants lamented on the absence of the health sector pre-pandemic. Many participants expressed difficulties engaging healthcare practitioners in early years meetings, community groups, and program planning. A lack of clarity as to the role of public health and primary health care in the early years and a lack of primary care providers was evident within many of the conversations. Significant service redesign in public health and primary health care across northern BC was believed to have influenced both short- and longer-term losses of connection between health care providers and early years community groups, often referred to as “early childhood tables”. One participant described that *“when public health left the early years table that was a loss”*. Participants expressed that this resulted in uncertainty as to what the healthcare sector, specifically the regional health authority, can provide or how health services support the early years work in northern BC. Some participants situated in smaller communities didn’t feel this same sense of loss, describing it was more difficult for healthcare services to leave or say no to supporting early years initiatives when there was an existing relationship outside of work roles. While healthcare was seen as a ‘*major player*’, education and child serving government ministries were also highlighted as significant partners in both day-to-day work, and drivers of change within community settings.

#### Support and autonomy

*Compassion is the thread that weaves this work together.* Across sectors, individuals emphasized they are in this work because they care about the children and families they work with. Compassion was described as a flexible practice that starts with listening deeply to what people are sharing, meeting people where they are at, and being inclusive and open to their diverse needs. There was consensus across participants that compassion starts within our teams, leadership, and workplaces. Participants explained when leaders were compassionate in organizations, it created a supportive culture that then translated into how they worked alongside families. Compassionate leadership was described as promoting a climate of trust and encouragement whereby those working together can become a cooperative and collaborative team, listen carefully to each other, empathize, work to understand the challenges, and help each other. Supportive and compassionate work environments recognized the staff as professionals equipped for the job but also as humans with lives outside of work dealing with personal demands. Some described that being in a smaller community lends itself well to the compassionate process, as relationships are diverse and dual, as you get to know people across their ‘role’ boundaries.

Participants spoke to autonomy as having the ability to make independent decisions in their work and direction of the services. One way this was expressed was in service changes during the COVID-19 pandemic where individuals expressed finally having the ability and decision-making power to be able to act on ideas and not carry on with the same way of providing services. Stories were shared of the initial disappointment and concern when programs were cancelled due to lockdowns and social distancing restrictions and the loss of connection with families. However, out of these emerged new ways to care for families and newfound autonomy for providers through the flexibility and rapid innovation that was required. The need to make decisions quickly bypassed slow processes or *“we’ve always done it this way*” attitudes which often prevented change prior to COVID-19. This autonomy fostered innovation. Some participants described that they had the courage to change and try new things because everyone was in a process of learning how to re-structure services. One example included establishing virtual programs which were now accessible across multiple communities. These virtual programs increased access to supports that some communities never had before the pandemic and staff were supported to provide this to families beyond their local community. Despite the challenges of the pandemic, many found it accelerated self-reflection, mindfulness, courage, innovation, and creativity in the early years sectors by giving individuals and teams options to try something new or apply traditional knowledge that may have been set aside as a means of restoring the ways things had been done in the past. Applying CSL principles and activities helped people to ‘step away’ and then re-engage in the work. This allowed for non-judgmental rest and recovery time, which resulted in the strengthening of supportive and effective team and inter-team working.

## Discussion

In facilitating a space that allowed for vulnerability and relational ways of engaging we discovered commitment and a willingness to consider new ideas and partnerships. The learnings from this event were twofold. While we gathered valuable learning about early years services and collaboration in northern BC that will support greater integration of early years services through shared priorities and efforts, we also had rich learning on applying CSL principles for the purpose of engagement and fostering innovation across sectors, and the use of an online, virtual space.

In early years work settings, the busy nature of the work can limit the perceived opportunity for mindfulness and self-compassion practices. The virtual asynchronous and synchronous approach allowed for the practice of mindfulness as a foundational element as participants were able to make thoughtful and informed decisions about when to attend, how to engage, and when to re-visit the asynchronous material provided in the Summit based on their own needs. We intentionally provided multiple avenues for engagement to allow individuals to be autonomous in how they chose to participate and engage. The online setting also allowed for individuals to listen to their physical and emotional needs in ways that in-person contexts might not allow. For example, individuals were encouraged to mindfully care for themselves during the day by turning their cameras off as needed, making a cup of tea, or stretching. Applying a CSL approach allowed a space for mindfulness to be transparent and encouraged by finally giving participants the time to work through what is happening in themselves and their local spaces. This was fostered through breathing breaks, reflection questions, and moments for celebration of individual work. The exercises allowed for the creation of fellowship, support, and attention to self – which then helped to bring people together and allow time for reflection in the ‘activities’ of the moment.

In our planning we looked to demonstrate the diversity and value of different perspectives by opening the Summit widely to participants with no restrictive eligibility criteria for registration. The transition to a virtual event also allowed us to broaden our reach and supported equitable opportunity for individuals to participate since no travel was required. The online nature created opportunity for participants to join from a wide range of physical spaces geographically, but also supported inclusion of less able-bodied individuals who may struggle with travel, new spaces and their accessibility, or feeling well enough to participate in person. This resulted in a diverse group. CSL looks to foster inclusion, by acknowledging that individuals arrive with diversity in culture, education, experience, and discipline (professional background). As an active process CSL recognises hierarchy exists yet concurrently works to dismantle this by creating and maintaining psychological safety and valuing diversity and positive attitudes to differences, to support a space where all voices can be heard. At the live workshop, the breakout facilitators played an important role to (1) ensure all participants were invited into the conversation and had opportunity to share their experiences, and (2) to create both a safe and brave space where participants could validate and challenge each other. CSL principles pay attention to uncomfortable, complicated, or charged conversations and open a space in which discomfort and disappointments between sectors can be shared. Not only did applying the CSL principles facilitate a safe space where individuals were supported to talk about their experiences, errors, problems, and uncertainties without fear of judgment [20]; it created a ‘brave space’ that actively encouraged dialogue and sharing of experiences to bring about new understandings. For example, participants felt brave enough to say who they felt was not showing up at the table, in this case, the health sector, but also encouraged dialogue on what could be done to change this. This brave space can support innovation and development of ideas for new and improved ways of conceptualizing and delivery programs and services [20].

The Summit objectives emphasized that this was a learning event with the purpose of building shared understanding and priorities for future work. By inviting individuals to share their knowledge and experiences of work happening to support children and families in the early years across the north and celebrate successes, the Summit promoted a culture of learning and exploration. Approaching it in this manner we found that participants were excited to be involved as the Summit offered a space where the surge of compassion, enthusiasm, and creativity in working in the early years was heard, understood, and celebrated. Literature on CSL and innovation describe that compassion involves creating space and freedom for individuals to experiment, discover and apply [20, 29, 30]. The shared experiences and vision of participants allowed for empathic conversations in the virtual space. Literature on the use of digital spaces cites shared experience as critical to facilitating empathic connections online [31]. Everyone who showed up to the Summit had an interest in healthy early childhood development and an assumed desire for change in this area. This created a common bond across the group and when they heard from other individuals who share similar experiences or feelings, this created a space to build relationships.

At the conclusion of the event, many participants expressed their responsibility in participating as an active process and shared a desire to remain connected and involved in future opportunities. Moving forward, participants spoke to the desire for knowledge exchange. Summit participants identified the value of an online knowledge hub that could house resources and local information both from and for communities. There was also interest in the development of a northern community of self-compassionate practice and leadership specifically focused on early childhood education, health, and wellness. A wise action was the idea of mentoring compassionate leaders and developing a ‘connection’ between champions in communities. It was proposed that a regional table with representatives from each sector could function as compassionate leaders in this work to deepen learning and share knowledge in ways that could transform the early years sectors vision of a ‘new blanket’. Ultimately, in applying CSL principles for engagement we began to develop collective, compassionate leadership to support a culture of improvement and innovation within and across organizations [20].

### Next steps

Following the Summit, the findings were shared with participants, the project advisory committee, and leaders across northern BC organizations. These groups were asked to identify further opportunities to share findings across their organizations. Building on the feedback from participants, the advisory committee is recruiting additional members, representative of sectors across the regions of northern BC to form a regional SEED table. This table will be responsible for bringing together leadership from across the north to further this work by refining regional priorities, exploring additional funding and research opportunities, and developing resources that can be used at the local level by champions to foster community multi-sectoral early childhood tables. Further, this group is focusing on how we address the ‘ranked’ needs (Fig. 1) with the themes we drew from the synchronous workshop during the Summit. The SEED BC website has been established as an ongoing knowledge hub where resources, local information for communities, as well as the materials from the Summit are stored and new opportunities can be shared by all partners from across our region.

The current patchwork approach to early years services described by participants notably creates gaps and leaves the onus of weaving together support on the family. This may leave many children and families without access to comprehensive services that would support healthy development and promote early learning, particularly before they enter the school system. Weaving a new blanket will require further communication, coordination, and collaboration across sectors to develop an integrated approach to supporting families and children in the early years, but also requires the engagement of families. This Summit was designed to engage providers and leaders in early years care and services. While some parent/caregivers or parent peer support workers participated and others expressed duality as a professional and parent, this was not solely a patient engagement focused event. We acknowledge it is critical to better understand patients’ (in this case children and primary caregivers) experiences as well. These voices need to be engaged in future work to support enhanced service delivery and to inform patient and provider education and policies [32].

### Limitations

Completion of the evaluation survey for the Summit was poor despite being distributed to all participants via email in the week following the Summit and re-distributed in a postcard reminder by mail. We anticipate many felt they already shared their feedback in the workshop reflection sessions but acknowledge this as a limitation. We also acknowledge that the voices of families were largely absent from this event other than participants who recognized their dual positionality as both care providers and parents/caregivers or grandparents. We recognized the value of including families but at this stage we did not have a network to ‘find them’ or advice on ‘how to best’ engage families meaningfully, respectfully, and with compassion. This project provided a starting conversation with those working directly with families to explore what family engagement could look like in the region and when and how best to involve busy families who are already often burdened with demands. This conversation is continuing with the regional advisory table which has since been established.

## Conclusions

Regardless of the population of interest, shifting from siloed work to collaboration and integration across sectors is complex. For any team or group CSL principles can facilitate a supportive space for individuals to gather and be vulnerable with a view to holding innovative conversations that could lead to systems change. This was also possible in an online, virtual space. In our context this was focused on beginning to understand ways to better support children and families in the early years in both inclusive and culturally aware ways. The opportunity to share and exchange information, feel listened to, and discover meaningful similarities and differences across services, geographies, cultures, and disciplines was a positive experience. The relational ways of being helped providers see different perspectives and in our experience in using this to come together across sectors we discovered commitment, and a willingness for those present to consider new ideas and partnerships that would allow for greater integration of early years services in northern BC. Within a CSL approach, personal mastery, reflective conversations, and systems thinking offer space for authentic interpersonal relationship building, a deep and shared understanding of how and where current systems are perpetuating tension and stress [33]. Taking care of self-and others allows us to shift away from the ‘cycle’ of doing things the same way and work towards different futures.

## Data Availability

Not applicable.

## Abbreviations

BC: British Columbia
CSL: Compassionate systems leadership
EDI: Early development instrument
SEED BC: Sharing to change early childhood experiences and promote healthy development in Northern BC

## References

[1] World Health Organization. Improving early childhood development: WHO Guideline. World Health Organization. 2020. https://www.who.int/publications/i/item/97892400020986. Accessed 19 Feb 2022. 32200595

[2] National Scientific Council on the Developing Child. Children’s emotional development is built into the architecture of their brains: Working paper no. 2. 2004. https://developingchild.harvard.edu/resources/childrens-emotional-development-is-built-into-the-architecture-of-their-brains/. Accessed 19 Feb 2022.

[3] Hertzman C (2013). The significance of early childhood adversity. Paediatr Child Health.

[4] Williams RC, Biscaro A, Clinton J (2019). Relationships matter: how clinicians can support positive parenting in the early years. Paediatr Child Health.

[5] Low BJ, Low MD (2006). Education and education policy as social determinants of health. AMA J Ethics.

[6] Avellar SA, Supplee LH (2013). Effectiveness of home visiting in improving child health and reducing child maltreatment. Pediatrics.

[7] Casillas KL, Fauchier A, Derkash BT, Garrido EF (2016). Implementation of evidence-based home visiting programs aimed at reducing child maltreatment: a meta-analytic review. Child Abus Negl.

[8] Peacock S, Konrad S, Watson E, Nickel D, Muhajarine N (2013). Effectiveness of home visiting programs on child outcomes: a systematic review. BMC Public Health.

[9] Sweet M, Appelbaum M (2004). Is home visiting an effective strategy? A meta-analytic review of home visiting programs for families with young children. Child Dev.

[10] Cannon JS, Kilburn MR, Karoly LA, Mattox T, Muchow AN, Buenaventura M. Investing early: taking stock of outcomes and economic returns from early childhood programs. Rand Corporation. 2017;10.7249/RR1993. Accessed 19 Feb 2022. 10.7249/rhq-07-04-06 PMC6075808 30083418

[11] Tran TD, Luchters S, Fisher J (2017). Early childhood development: impact of national human development, family poverty, parenting practices and access to early childhood education. Child Care Health Dev.

[12] Antonelli RC, McAllister JW, Popp J. Making care coordination a critical component of the pediatric health system: a multidisciplinary framework. The Commonwealth Fund. 2009. http://www.commonwealthfund.org. Accessed 19 Feb 2022.

[13] Council on Children with Disabilities and Medical Home Implementation Project Advisory Committee, Turchi RM, Antonelli RC, Norwood KW, Adams RC, Brei TJ, Burke RT, Davis BE, Friedman SL, Houtrow AJ, Kuo DZ, Levy SE, Wiley SE, Kalichman MA, Murphy NA, Cooley WC, Jeung J, Johnson B, Klitzner TS, Lail JL, Lindeke LL, Mullins A, Partridge L, Schwab W, Stille C, Waldron D, Wells N, Sia C. Patient- and family-centered care coordination: a framework for integrating care for children and youth across multiple systems. Pediatrics. 2014;133(5):e1451–60. 10.1542/peds.2014-0318. 10.1542/peds.2014-0318 24777209

[14] Gerlach A, Gulamhusein S, Varley L, Perron M (2021). Structural challenges & inequities in operating urban indigenous early learning and child care programs in British Columbia. J Child Stud.

[15] Kania J, Kramer M. Embracing emergence: how collective impact addressing complexity. Stanford Soc Innov Rev. 2013;(1):1–7. 10.48558/ZJY9-4D87.

[16] First Nations Health Authority. About: Northern. 2019. http://www.fnha.ca/about/regions/north. Accessed 19 Feb 2022.

[17] Janus M, Offord DR (2007). Development and psychometric properties of the early development instrument (EDI): a measure of children’s school readiness. Can J Behav Sci.

[18] The Human Early Learning Partnership. Early development instrument British Columbia, 2016–2019 wave 7 provincial report. University of British Columbia. 2019. http://earlylearning.ubc.ca/media/edibc_wave7_2019_provincialreport.pdf. Accessed 19 Feb 2022.

[19] Schroeder J, Rowcliffe P. Growing compassionate systems leadership: a Tookit. The Human Early Learning Partnership. 2019. http://earlylearning.ubc.ca/media/systems_toolkit_2019_final.pdf. Accessed 19 Feb 2022.

[20] West M, Eckert R, Collins B, Chowla R. Caring to change: how compassionate leadership can simulate innovation in health care. The King's Fund. 2017. https://www.kingsfund.org.uk/sites/default/files/field/field_publication_file/Caring_to_change_Kings_Fund_May_2017.pdf. Accessed 19 Feb 2022.

[21] SEED BC. https://seedbc.ca/ (2020). Accessed 19 Feb 2022.

[22] Northern Health Indigenous Health. 5 facts about indigenous people in Northern BC [Internet]. Northern Health. [cited 2022 Sep 30]. Available from: https://www.indigenoushealthnh.ca/sites/default/files/2019-06/facts-about-indigenous-people.pdf

[23] Halseth R, Greenwood M. Indigenous early childhood development in Canada: Current state of knowledge and future directions. National Collaborationg Centre for Aboriginal Health. 2019. https://www.nccih.ca/en/. Accessed 19 Feb 2022.

[24] Truth and Reconciliation Commission of Canada. Truth and reconciliation commission of Canada: Calls to Action. 2015. https://www2.gov.bc.ca/assets/gov/british-columbians-our-governments/indigenous-people/aboriginal-peoples-documents/calls_to_action_english2.pdf. Accessed 19 Feb 2022.

[25] Gerlach AJ, Browne AJ, Suto MJ (2016). Relational approaches to fostering health equity for indigenous children through early childhood intervention. Heal Sociol Rev.

[26] Browne AJ (2017). Moving beyond description: closing the health equity gap by redressing racism impacting indigenous populations. Soc Sci Med.

[27] Wright A, Wahoush O, Ballantyne M, Gabel C, Jack SM (2018). Selection and use of health services for infants’ needs by Indigenous mothers in Canada: integrative literature review. Can J Nurs Res.

[28] Braun V, Clarke V, Hayfield N, Terry G, Liamputtong P (2019). Thematic analysis. Handbook of research methods in health social sciences.

[29] West MA, Galiana L, Sanso N (2019). Compassionate leadership in health and care settings. The power of compassion.

[30] West MA, Montgomery A, van der Doef M, Panagopoulou E, Leiter MP (2020). Compassionate and collective leadership for cultures of high-quality care. Connecting healthcare worker well-being, patient safety and organisational change: the triple challenge.

[31] Hargreaves S, Bath PA, Duffin S, Ellis J (2018). Sharing and empathy in digital spaces: qualitative study of online health forums for breast cancer and motor neuron disease (amyotrophic lateral sclerosis). J Med Internet Res.

[32] Bombard Y, Baker GR, Orlando E, Fancott C, Bhatia P, Casalino S, Onate K, Denis J, Pomey MP (2018). Engaging patients to improve quality of care: a systematic review. Implement Sci.

[33] Rowcliffe P, Schroeder J. Compassionate systems leadership: leveraging disruption for transformative change [Internet]. EdCan Network. 2021 [cited 2022 Sep 29]. Available from: https://www.edcan.ca/articles/compassionate-systems-leadership-2/

